# SPBP Is a Sulforaphane Induced Transcriptional Coactivator of NRF2 Regulating Expression of the Autophagy Receptor p62/SQSTM1

**DOI:** 10.1371/journal.pone.0085262

**Published:** 2014-01-09

**Authors:** Sagar Ramesh Darvekar, Julianne Elvenes, Hanne Britt Brenne, Terje Johansen, Eva Sjøttem

**Affiliations:** Molecular Cancer Research Group, Department of Medical Biology, University of Tromsø, Tromsø, Norway; National University of Singapore, Singapore

## Abstract

Organisms exposed to oxidative stress respond by orchestrating a stress response to prevent further damage. Intracellular levels of antioxidant agents increase, and damaged components are removed by autophagy induction. The KEAP1-NRF2 signaling pathway is the main pathway responsible for cell defense against oxidative stress and for maintaining the cellular redox balance at physiological levels. Sulforaphane, an isothiocyanate derived from cruciferous vegetables, is a potent inducer of KEAP1-NRF2 signaling and antioxidant response element driven gene expression. In this study, we show that sulforaphane enhances the expression of the transcriptional coregulator SPBP. The expression curve peaks 6–8 hours post stimulation, and parallels the sulforaphane-induced expression of NRF2 and the autophagy receptor protein p62/SQSTM1. Reporter gene assays show that SPBP stimulates the expression of p62/SQSTM1 via ARE elements in the promoter region, and siRNA mediated knock down of SPBP significantly decreases the expression of p62/SQSTM1 and the formation of p62/SQSTM1 bodies in HeLa cells. Furthermore, SPBP siRNA reduces the sulforaphane induced expression of NRF2, and the expression of the autophagy marker protein LC3B. Both these proteins contain ARE-like elements in their promoter regions. Over-expressed SPBP and NRF2 acts synergistically on the p62/SQSTM1 promoter and colocalize in nuclear speckles in HeLa cells. Collectively, these results suggest that SPBP is a coactivator of NRF2, and hence may be important for securing enhanced and sustained expression of NRF2 induced genes such as proteins involved in selective autophagy.

## Introduction

Oxidative stress causes damage to multiple cellular molecules, and is a major contributing factor in a variety of human diseases such as cancer, neurodegenerative disorders, inflammatory diseases, cardiovascular disease and ageing [1]. Cells have developed a defence system, a variety of antioxidant enzymes and molecules, to detoxify oxidative species. The transcription factor NRF2 (nuclear factor erythroid 2-related factor) is a master regulator of response to oxidative stress, regulating the basal and inducible expression of many antioxidant pathway genes containing antioxidant response elements (AREs) in their transcription control region (reviewed in [1], [2]). NRF2 knock-out mice display increased sensitivity to a number of xenobiotics, thus highlighting the importance of NRF2 in cellular stress responses (reviewed in [3], [4]). In unstressed conditions, the Cullin3-adaptor protein KEAP1 constitutively targets NRF2 for ubiquitin conjugation and degradation by the proteasome. Post-translational modification of KEAP1 and NRF2 by electrophiles and oxidants impairs the interaction between KEAP1 and NRF2, resulting in stabilisation and rapid accumulation of NRF2 in the nucleus [1], [5]. Here, NRF2 transactivates the antioxidant response element (ARE) present in the promoter region of many antioxidant genes. Constitutively activated NRF2 promotes longevity and confers increased tolerance to oxidative stress in model organisms [6], [7]. Sulforaphane, a naturally occurring isothiocyanate derived from cruciferous vegetables, stimulates induction of enzymes involved in xenobiotic metabolism [8], [9] and proteasome subunit levels via an NRF2-dependent mechanism [10].

Autophagy is an essential cellular mechanism of adaption to external or internal stress. It includes degradation of intracellular components during starvation conditions, elimination of aggregated proteins, turnover of damaged or old organelles, and protection against invading microorganisms (reviewed in [11]). Autophagy can mediate cardioprotection and neuroprotection, delay the pathogenic manifestations of ageing and prolong life span (reviewed in [12]). The autophagic process is initiated by formation of a double membrane structure, the autophagosome, that grows and isolates a part of the cytosol. The autophagosome matures and fuses with a lysosome, leading to degradation of the autophagosomal contents. An essential step in autophagy is the conjugation of phosphatidylethanolamine to microtubule-associated protein 1 light-chain 3 (LC3). This converts the soluble form of LC3 (LC3 I) to the LC3 II form that specifically associates with autophagosomes [13]–[15]. p62/SQSTM1 (hereafter termed p62) acts as a receptor for selective autophagy, recognising the LC3 II protein in the autophagic membrane and ubiquitin molecules attached to the autophagic substrate determined for degradation [16], [17]. Accumulation of p62 very often reflects a transient or constitutive inhibition of autophagy. Brain-specific block in autophagy in mice causes rapid development of neurodegeneration accompanied by accumulation of p62 in ubiquitinated protein inclusions [18], [19]. In the heart, cardiac-specific deficiency in autophagy causes myopathy and contractile dysfunction accompanied by accumulation of ubiquitin and p62 [20]. Increased levels of p62 correlate with aggressive breast cancer [21] and prostate cancer [22], and a study suggests that accumulation of p62 may have a strong tumor promoting effect [23]. p62 is also a scaffold protein for cell survival and death signalling pathways, and it is assumed that accumulation of p62 leads to dysregulated activation of these signalling pathways (reviewed in [24]). Stress signals generated in cells during inflammation, protein misfolding and aggregation, oxygen and UVA exposure, or exposure to drugs like arsenic, resveratrol, PMA and valproic acid, are shown to induce p62 transcription. Conversely, p62 expression is found to be down-regulated when cells are exposed to amino acid starvation and hypoxia-activated autophagy. Recently it was shown that sulforaphane induces p62 expression in an NRF2 dependent manner. NRF2 binds to an ARE element in the p62 promoter and in this way enhances p62 expression. p62, on the other hand, competes with NRF2 for KEAP1 binding leading to NRF2 stabilisation and accumulation. In this manner p62 generates a positive feedback loop contributing to prolonged activation of NRF2 in response to oxidative stress [25].

**Figure 1:**
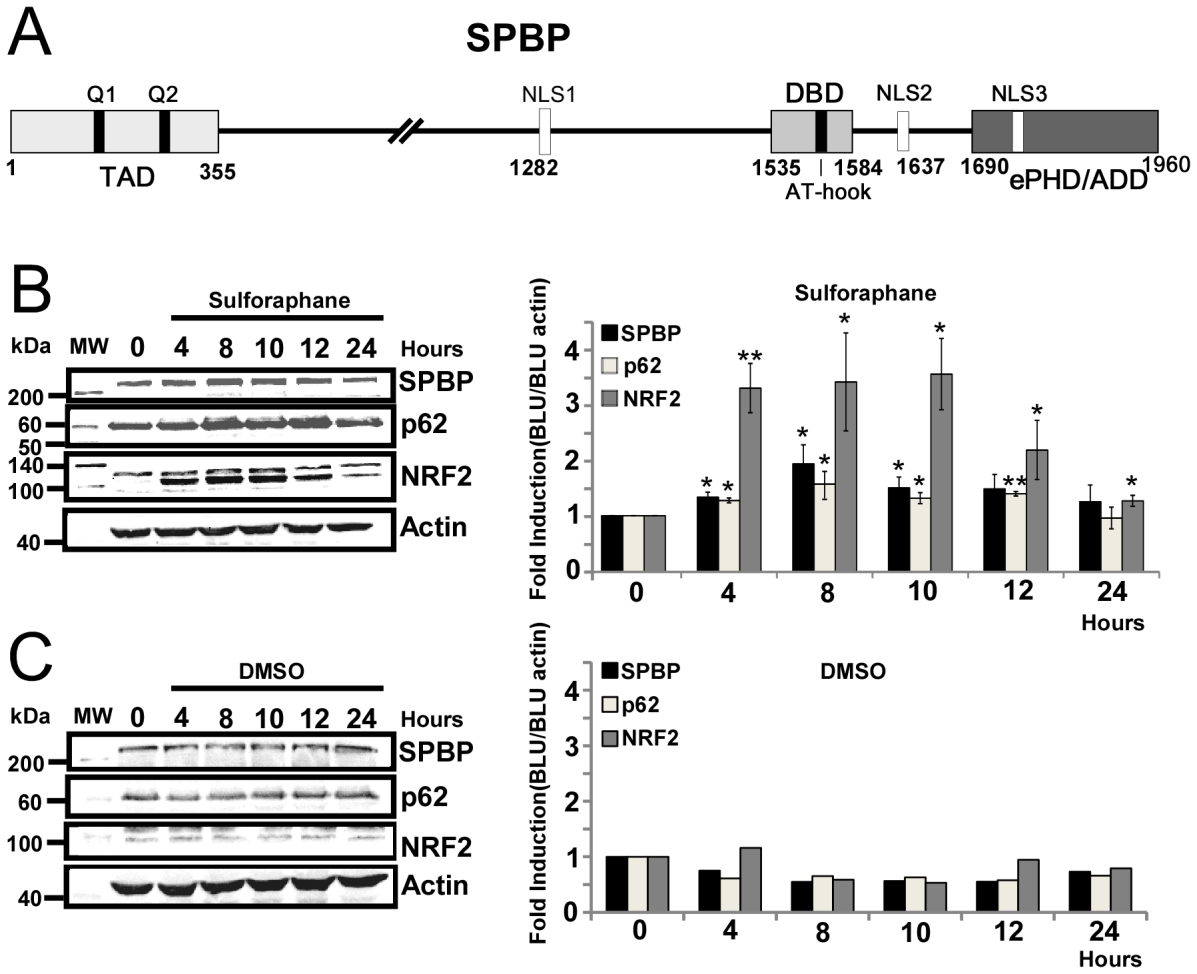
Sulforaphane enhances SPBP expression. (**A**) Schematic representation of the domain structure of human SPBP. TAD: trans-activation domain, DBD: DNA binding domain, NLS: Nuclear Localisation Signal, ePHD/ADD: extended PHD/ADD domain, Q1/Q2: Glutamine rich stretches. Numbers below indicate amino acid positions. (**B**) NRF2, SPBP and p62 display similar induction upon sulforaphane treatment. HeLa cells were exposed to 20 µM sulforaphane and cell extracts harvested for the indicated time points. Equivalent aliquots from the extracts were subjected to SDS-PAGE and western blot using specific anti-SPBP antibody, anti-p62 antibody, anti-NRF2 antibody or anti-actin as indicated. Fold induction calculated and correlated to actin in three independent experiments with standard deviations are shown to the right (**p<0.01, *p<0.05). (**C**) Control experiment showing that DMSO alone does not induce any changes in NRF2, SPBP or p62 expression levels. Equivalent aliquots of HeLa cell extracts exposed to DMSO and harvested for the indicated time points were subjected to SDS-PAGE and western blot using specific anti-SPBP antibody, anti-p62 antibody, anti-NRF2 antibody or anti-actin antibody as indicated. Fold induction calculated and correlated to actin are shown to the right.

The transcriptional coregulator SPBP (also named TCF20) is a 220 kDa multidomain nuclear protein expressed in most cells and tissues (Figure 1A) [26], [27]. SPBP was originally identified as a platelet-derived-growth-factor (PDGF) induced protein involved in transcriptional activation of the matrix metalloprotease-3 (MMP3) promoter [28]. This activation was later found to be dependent on Ets1 binding to the MMP3 promoter [27]. SPBP is also reported to enhance the transcription potential of Sp1 and c-Jun [26], androgen receptor [29], and SNURF/RNF4 [30]. SPBP associates strongly with nucleosomes via two separate nucleosome binding domains [31]. One is the C-terminal cysteine rich region with similarity to an extended Plant Homeo Domain/ATRX-DNMT3L-DNMT3A domain (ePHD/ADD)(Figure 1A) and the other is a novel domain localized adjacent to the DNA binding domain with a conserved AT-hook motif [26]. The ePHD/ADD domain is also involved in protein-protein interactions [27]. In the cell nucleus, SPBP displays relatively low mobility and is enriched in chromatin dense regions, clearly indicating that it is a chromatin binding protein [31].

Here we have shown that expression of the transcriptional coregulator and chromatin binding protein SPBP is induced by sulforaphane. Sulforaphane is a potent inducer of ARE-driven gene expression via KEAP1-NRF2 signalling. The autophagic receptor and signalling scaffold protein p62 contains conserved ARE elements in its promoter region, and we found that SPBP enhances p62 expression via these elements. SPBP associated with the p62 promoter, and siRNA mediated knock down of SPBP significantly downregulated p62 expression and formation of p62 bodies in HeLa cells. Furthermore, knock down of SPBP affected expression of LC3B, and the sulforaphane induced expression of NRF2. NRF2 regulates its own expression via ARE elements in its promoter region, and an ARE-like element is also present in the LC3B promoter. Hence, SPBP acts as a transcriptional coactivator of NRF2, and may be important for enhanced and sustained cellular response to oxidative stress mediated by KEAP1-NRF2 signalling, including the NRF2 mediated induction of proteins involved in selective autophagy.

## Materials and Methods

### Plasmid Constructs

cDNA constructs were subcloned into Gateway entry vectors and expression clones made as described (Invitrogen). All constructs were verified by DNA sequencing. Human NRF2 was amplified by PCR using primers 5′-GGGGACAAGTTTGTACAAAAAAGCAGGCTCCACCATGATGGACTTGGAGCTGCCG-3′ and 5′-GGGGACCACTTTGTACAAGAAAAGCTGGGTCTACCTAGTTTTTCTTAACATCTGGC -3′ and image clone IRAUp969G0565D as template, and recombined into pDONR221 (Invitrogen). The −1781/+46 p62 promoter construct, the −1781/+46 construct with a mutated ARE site at position −1300, and the NQO1 promoter constructs are described previously [25]. The ARE site at position −330 was mutated using primer 5′-CAACTGAGGATATTGCAGGGACATGGCCAGGCCCAAGC-3, and the AP1 binding site was mutated using primer 5′-CGGGCTCGAGATCTCTCTGTCACTGCCGCCAGACC-3′. All mutation constructs were generated by PCR using Pfu Turbo polymerase (STRATAGENE) and verified by DNA sequencing with BigDye v3.1 (Applied Biosystems). The SPBP constructs are described previously [27]. The TpT and 601 nucleosome position sequences, and the Control sequence, were amplified by PCR using primers 5′-CGCGAAGCTTCTCCTGCAGACGCGTCGGTGTTAGAGCC-3′ and 5′-CGCGCTGCAGTCTAAGCTTGAATTCTCTAGACAGTGTCCC-3′ for TpT, 5′-CGCGAAGCTTCTCCTGCAGCCTGGAGAATCCCGGTGCCG-3′ and 5′-CGCGCTGCAGTCTAAGCTTCACAGGATGTATATATCTGAC-3′ for 601, and 5′-CGCGAAGCTTCTCCTGCAGTAAAAGATGCTGAAGATC-3′ and 5′-CGCGCTGCAGTCTAAGCTTATAATACCGCGCCACATAGC′ for Control. Plasmids pTpT and p601, kind gifts from R. Kingston's group, were used as template. The PCR products were digested by *HindIII*, inserted into the *HindIII* sites of the p62 and NQO1 promoter constructs, and verified by DNA sequencing.

### Cell Culture

HeLa (ATCC CCL2) and HEK293 (ATCC CRL-1573) cells were grown in Dulbecco's modified Eagle's medium (DMEM). U2OS-TA cells [27] were maintained in DMEM with 400 µg/ml geneticin (G418, SIGMA) and puromycin dihydrochloride (1 µg/ml). In addition, the media for all cell lines were supplemented with 10% fetal bovine serum (Biochrom AG, S0615) and 1% streptomycin-penicillin (Sigma, P4333). Cultured cells were maintained at 37°C with 95% air and 5% CO_2_ in a humidified atmosphere.

Cells were amino acid starved in Hanks Balanced Salts medium (HBSS)(H137, SIGMA) for 2 hours before treatment for 4 hours with the following pharmacological agents Bafilomycin A1 (0.2 µM), Sulforaphane (20 µM), or MG132 (0.2 µM).

### Antibodies and Reagents

The following primary antibodies were used: Rabbit anti-SPBP antibody [27], monoclonal anti-p62 antibody (BD Transduction Laboratories), rabbit anti-NRF2 antibody (Santa Cruz, SC-13032, Abcam ab62352), rabbit anti-LC3B antibody (SIGMA, L7543), rabbit anti-KEAP1 antibody (ProteinTech, 10503-2-AP), mouse anti-Flag antibody (Stratagene, 200471), rabbit anti-actin antibodies (Sigma, A 2066). Secondary antibodies used were: HRP-conjugated goat anti-rabbit IgG and anti-mouse IgG antibodies (BD Bioscience Pharmingen). AlexaFluor 568 and AlexaFluor 548 conjugated goat anti mouse and anti-rabbit IgG (Molecular Probes). Sulforaphane (S 4441), Bafilomycin A1 (B 1793) and MG 132 (C 2211) were purchased from Sigma-Aldrich.

### Western Blotting with total cell extracts

Western Blotting using total cell extracts were performed essentially as described previously [25]. For siRNA knock-down, the cells were transfected with 20 nM of each siRNA using RNAiMAX (Invitrogen). The SPBP, p62 and Control siRNAs are described previously [25], [27]. The cells were harvested 48–72 hours post transfection. Sulforaphane, Bafilomycin A1 and MG 132 treatments were as indicated in each figure. Quantitation of bands were performed using the LumiAnalyst™ Image Analysis Software, and all bands were correlated to actin blotted on the same membrane.

### Reporter gene assays

Subconfluent HEK293 cells in 24-well tissue culture dishes (Becton Dickinson) were transiently transfected using Metafectene Pro (Biontex) according to the manufacturer's protocol. All wells were cotransfected with 60 ng of luciferase reporter vectors, 100 ng or as indicated in the figures of SPBP expression vector (pDEST-HA-SPBP), 100 ng of CBP expression vector (pHA-CBP), 50 ng or as indicated in the figure of pDEST-HA-NRF2 and 5 ng of a β-galactosidase expression vector. pcDNA_3_HA (Invitrogen) was used to equalize the concentration of DNA in each transfection. Extracts were prepared 20 hours post transfection and analyzed essentially as described previously [27]. All assays were performed in triplicates and repeated at least three times.

### Chromatin Immunoprecipitation

Chromatin Immunoprecipitation was performed mainly as described [27]. 1.5×10^7^ HeLa cells were used for each tested condition. Crosslinking with 1% formaldehyde was carried out for 10 minutes at 25°C. This was stopped by adding 0.125 M glycine for 5 minutes, 25°C. Harvested cells were resuspended in 500 µl Lysis Buffer (1% SDS, 10 mM EDTA, 50 mM Tris-HCl pH 8.1, protease inhibitor cocktail), and incubated 10 minutes on ice before sonication 20 minutes in a Bioruptor (Diagenode). The DNA fragments were purified using a QIAquick Spin Kit (QIAGEN). One to five µl from a 30 µl DNA extraction were used for 35 cycles of PCR amplification. PCR primers used to amplify the p62 promoter and the Cathepsin D promoter were as described [25].

### Real-Time PCR

Subconfluent HeLa cells in 6 well dishes were transfected with 20 nM SPBP siRNA or scrambled siRNA [27] using RNAiMAX (Invitrogen). 48–72 hours later RNA was isolated using RNAeasy Plus Minikit (QIAGEN), cDNA made using Transcriptor Universal cDNA Master (Roche), and RT-PCR performed on a STRATAGENE x 300 amplification system using FastStart Universal SYBR Green Master (Roche). Primers were p62: 5′-GGAGAAGAGCAGCTCACAGCCA-3′ and 5′-CCTTCAGCCCTGTGGGTCCCT-3′; Actin: 5′-TGACGGTCAGGTCATCACTATCGGCAATGA-3′ and 5′-TTGATCTTCATGGTGATAGGAGCGAGGGCA-3′; and GADPH: 5′-TGGGTGTGAACCACGAGAA-3′ and 5′-GGCATGGACTGTGGTCATGA-3′. The reactions were run for an initial step at 95°C for 10 min, followed by 40 cycles of amplification at 95°C for 15 s and 60°C for 1 min. All data were collected during the extension step, and a melting curve was obtained at the end of the PCR reaction to verify that only one product was produced. The calculations were carried out using Delta-delta CT method.

### Confocal microscopy

HeLa cells were cultured in 8-chambered cover slides (Nunc) and transiently transfected with 20 nM siRNA, or 50 ng pDestEGFP-NRF2 and 150 ng pDestCherry-SPBP using TransIT-LT1 (Mirus Bio) according to the manufacturer's protocol. Live cell images were taken one day post transfection using a Leica SP5 or a LSM510-META confocal laser scanning microscope. For quantification of p62 bodies, siRNA transfected cells were fixed in 4% paraformaldehyde 2 days post transfection. The cells were permeabilised by 0.1% Triton X-100, for 5 min at room temperature. Staining with antibodies was as described previously [32]. Images were obtained using a LSM510-META confocal microscope and p62 bodies were counted manually. Pearson's colocalisation coefficient and scatter were generated using Volocity (Perkin Elmer). Images were processed using Canvas version 11 (ACD systems).

### Coimmunoprecipitation

Subconfluent HeLa cells in 10 cm dishes (Nunc) were transiently co-transfected with either pDestEGFP-NRF2 and pDESTMyc-SPBP, or pDEST-EGFP and pDESTMyc-SPBP using Metafectene Pro (Biontex) according to the manufacturer's protocol. The cells were harvested 20 hours post transfection and co-immunoprecipitation performed using Chromotek-GFP-Trap (Allele) according to the manufacturer's protocol.

### Statistical analysis

Data were processed in Excel (Microsoft Corporation) to generate bar charts and perform statistical analyses. Student's t-test was performed for each dependent variable. *: p< = 0.05 was considered statistically significant, and **: p< = 0.01, very significant. p>0.05 was considered not significant (*n.s*.)

## Results

### SPBP expression is enhanced in parallel to NRF2 and p62 upon sulforaphane treatment

SPBP was originally described as a transcription factor induced when serum-starved NIH3T3 fibroblast cells were stimulated by PDGF or serum [28]. Since then, we and others have found SPBP to act as a transcriptional coactivator on several promoters [26]–[30], but also as a transcriptional corepressor [33]. In an attempt to identify the biological function of SPBP, we analyzed whether the expression level of SPBP was regulated due to specific cellular stimuli or stressors. Interestingly, we found that SPBP expression is induced upon sulforaphane treatment of HeLa cells (Figure 1B). Sulforaphane activates the redox sensitive transcription factor NRF2 inducing expression of detoxifying phase-II enzymes, such as quinone reductase and glutathione *S*-transferase, and has an inhibitory effect on histone deacetylase activity [8]. The sulforaphane induced SPBP expression increased and reached a peak level around 8 hours post sulforaphane treatment (Figure 1B). This expression pattern parallels the sulforaphane induction of NRF2 and of the NRF2 regulated selective autophagy receptor and signaling scaffold protein p62 (Figure 1B). DMSO alone did not affect the expression levels of SPBP, p62 and NRF2 (Figure 1C), clearly indicating that the enhanced expression of SPBP is a sulforaphane induced cellular response.

### SPBP enhances p62 promoter activity via ARE elements

**Figure 2:**
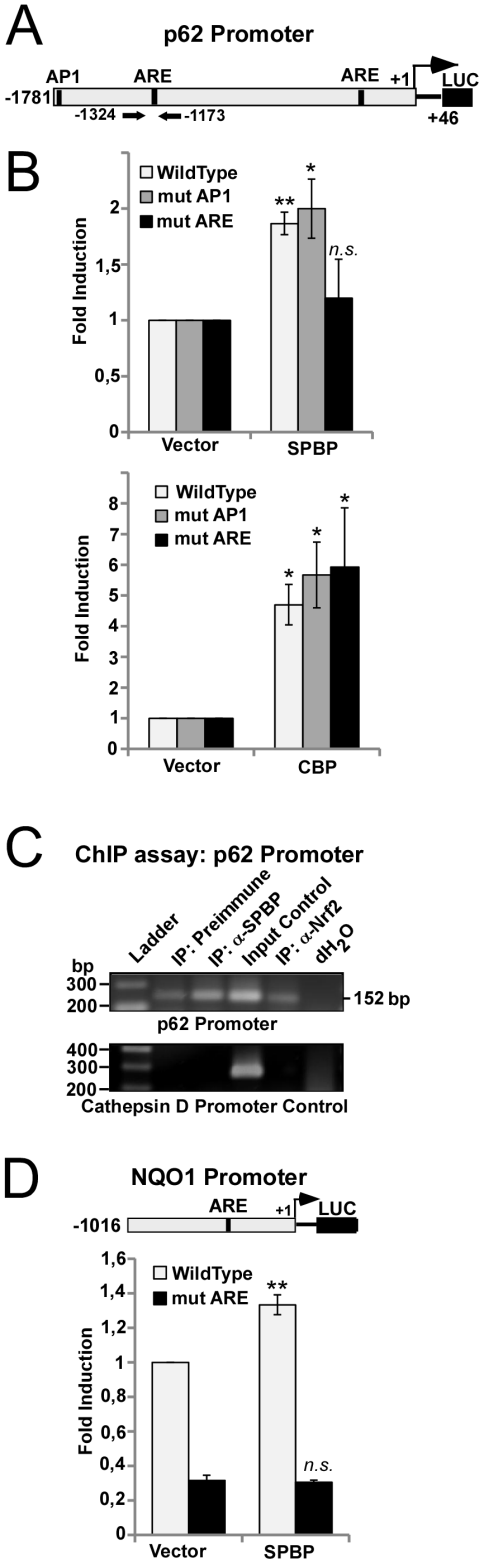
SPBP mediates transcriptional activation via ARE elements. (**A**) Schematic representation of the human −1781/+46 p62 promoter construct in front of the Luciferase gene. Conserved transcription factor binding sites relevant for this study are indicated. (**B**) Mutation of the ARE elements impairs SPBP mediated activation of the p62 promoter. Transient transfections were carried out in HEK293 cells using 60 ng of wild-type or mutated reporter vectors, and 100 ng of expression vectors for SPBP (upper panel) or CBP (lower panel). The data represent the mean of three independent experiments with standard deviations, each performed in triplicate (**p<0.01, *p<0.05, *n.s.* not significant). (**C**) Chromatin immunoprecipitations show that SPBP is associated with the p62 promoter. HeLa cell extracts (1.5×10^7^ cells per antibody) were immunoprecipitated with preimmune serum, polyclonal anti-SPBP antibody or polyclonal anti-NRF2 antibody. Input Control (1:40) was included. PCR was performed on chromatin precipitated with each antibody using primers aligning to positions −1324/−1173 in the p62 promoter (upper panel). Primers aligning to positions −3351/−3069 of the cathepsin D promoter were used as control. (**D**) SPBP mediated enhancement of the NQO1 promoter is dependent on the ARE element. Transient transfections were carried out in HEK293 cells using 60 ng of wild-type or mutated reporter vectors, and 100 ng of SPBP expression vectors. The data shows the mean of three independent experiments with standard deviations, each performed in triplicate (**p<0.01, *n.s.* not significant).

We have shown that sulforaphane induced expression of p62 is mediated by NRF2 binding to a conserved ARE element at position −1305/−1293 in the p62 promoter [25]. The similar sulforaphane induced expression of SPBP, NRF2 and p62, together with the transcriptional coactivator function of SPBP, prompted us to investigate whether SPBP could affect p62 promoter activity. Luciferase reporter gene assays using the p62 (−1781/+46) promoter [25] in front of the luciferase gene displayed a 2-fold induction of the p62 promoter mediated by SPBP over-expression (Figure 2B). The well characterized coactivator CBP enhanced p62 promoter activity 5-fold. CBP is shown to acetylate NRF2 as an antioxidant response, augmenting NRF2 DNA binding activity and also transcriptional activity [34]. To determine whether SPBP mediates its effect on the p62 promoter via NRF2, point mutations were introduced into two conserved ARE elements in the promoter, the previously reported NRF2 binding site at position −1305/−1293 and an additional conserved ARE element at position −330 which, according to ENCODE (http://genome.ucsc.edu), is found to be associated with NRF2 (Figure 2A). Mutation of the ARE element at −330 had significant impact on the basal p62 promoter activity (data not shown), suggesting that this ARE element is important for basal p62 promoter activity, while the −1330 site is the major site for sulforaphane mediated induction of the promoter [25]. In another p62 promoter construct, mutations were introduced into the AP1 binding site at position −1750 previously shown to be involved in NF-kB mediated expression of p62 [35]. Reporter gene assays showed that mutations of the ARE elements abolished the SPBP mediated induction of the p62 promoter (Figure 2B), while the CBP mediated activation seemed to be unaffected indicating that CBP may act on the p62 promoter via other transcription factor binding sites. This is in line with reports describing CBP as a coactivator of numerous transcription factors (reviewed in [36]). Chromatin immunoprecipitations were performed to evaluate whether endogenous SPBP was associated with the p62 promoter. Results presented in Figure 2C show that SPBP is associated with the p62 promoter. To further confirm that SPBP may mediate promoter activation via ARE sites, wild-type and ARE-mutated constructs of the NRF2 target gene promoter NQO1 were subjected to reporter gene assays with SPBP over-expression. SPBP induced a modest but significant 1.4 fold induction of the wild-type NQO1 promoter, while no induction of the ARE mutated NQO1 construct were observed (Figure 2D). These results clearly suggest that SPBP is able to enhance promoter activity mediated via ARE elements.

### Knock-down of SPBP impairs p62 expression and formation of p62 bodies

**Figure 3:**
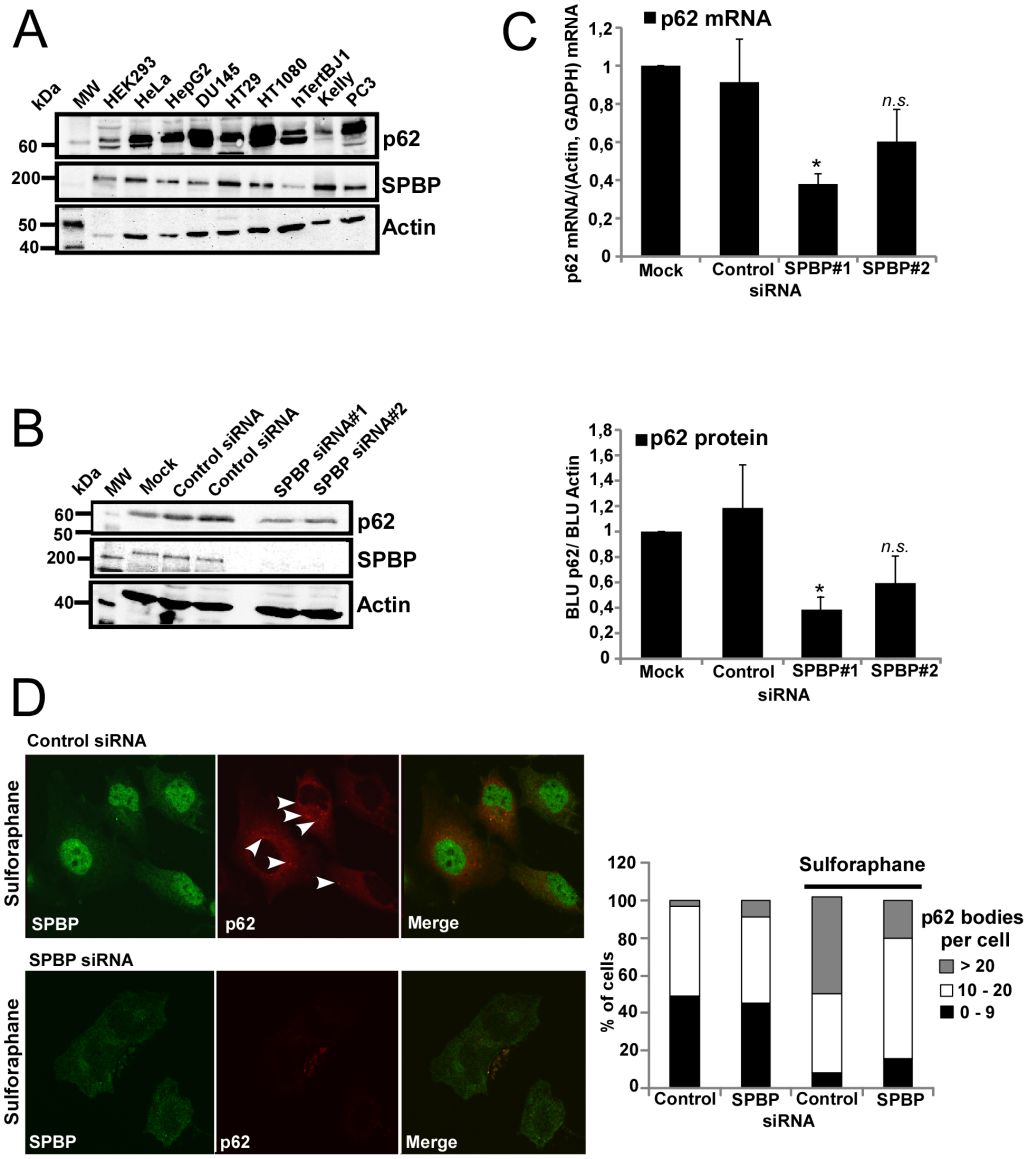
Knock down of SPBP impairs p62 expression and sulforaphane induced p62 body formation. (**A**) Endogenous SPBP and p62 are coexpressed in several cell lines. Extracts of the indicated human cell lines were analysed by western blotting using the indicated antibodies. (**B**) siRNA mediated knock down of SPBP reduces p62 expression level. HeLa cells transfected with two various SPBP siRNAs were analysed by western blotting using antibodies as indicated (left panel). The graph (right panel) shows the fold reduction calculated and correlated to actin in three independent experiments with standard deviations (*p<0.05, *n.s.* not significant). (**C**) Knock down of SPBP reduces the amount of p62 mRNA transcripts. The p62 mRNA levels were measured by quantitative RT-PCR. Hela cells were transfected with SPBP siRNAs or Control siRNA. RT-PCR reactions were run on p62, GADPH and β-actin mRNA. The average amount of p62 mRNA correlated to β-actin and GADPH mRNA based on two independent experiments are shown with standard deviations (*p<0.05, *n.s.* not significant). (**D**) p62 body formation upon sulforaphane treatment is reduced in SPBP siRNA knock down cells. HeLa cells were transfected with SPBP siRNA or Control siRNA and treated with 20 µM sulforaphane for 8 hours two days post transfection. Cells were fixed, stained with polyclonal antibodies against SPBP (green) and p62 (red) and analysed by confocal microscopy. The graph shows counting of p62 bodies in cells, each based on counting of more than 60 cells. Arrowheads indicate some of the p62 bodies in the cytoplasm.

SPBP and p62 is coexpressed in most human cells (Figure 3A) and tissues (www.proteinatlas.org). However, there is no clear correlation of their expression levels. To investigate whether SPBP may impact on the p62 expression levels in cells, siRNA mediated knock-down of SPBP was introduced into HeLa cells. Knock-down of SPBP using two different SPBP siRNAs [27] reproducibly decreased the amount of p62 (Figure 3B). Quantitative real-time PCR of p62 mRNA isolated from the SPBP siRNA treated HeLa cells showed a p62 mRNA reduction similar to the reduction of the p62 protein, indicating that SPBP affects p62 transcription (Figure 3C). This correlates well with our data showing that SPBP enhances p62 transcription via ARE elements in the p62 promoter. The p62 protein is both a scaffold protein in signalling pathways and a cargo receptor for selective autophagy (reviewed in [11], [37]).When cells are exposed to oxidative stress, the amount of p62-containing cytoplasmic bodies increases. Here we wanted to determine whether SPBP could have an effect on the stress-induced formation of p62 bodies. HeLa cells subjected to SPBP siRNA or Conrol siRNA, and sulforaphane were stained with antibodies against SPBP and p62. The images in Figure 3D (upper panel) show that sulforaphane stimulated HeLa cells display relatively high p62 expression levels forming distinct round p62 bodies in addition to a diffuse cytoplasmic distribution. Knock down of SPBP clearly impaired the formation of p62 bodies and diminished the cytoplasmic p62 staining (Figure 3D, lower panel). Quantitation of p62 bodies showed that knock down of SPBP by siRNA clearly reduced the formation of p62 bodies in sulforaphane treated cells compared to control siRNA (Figure 3D, right graph). Collectively these results suggest that SPBP plays a role in the p62 mediated oxidative stress response.

### SPBP does not enhance NRF2 mediated transcription by facilitating nucleosome traversal

**Figure 4:**
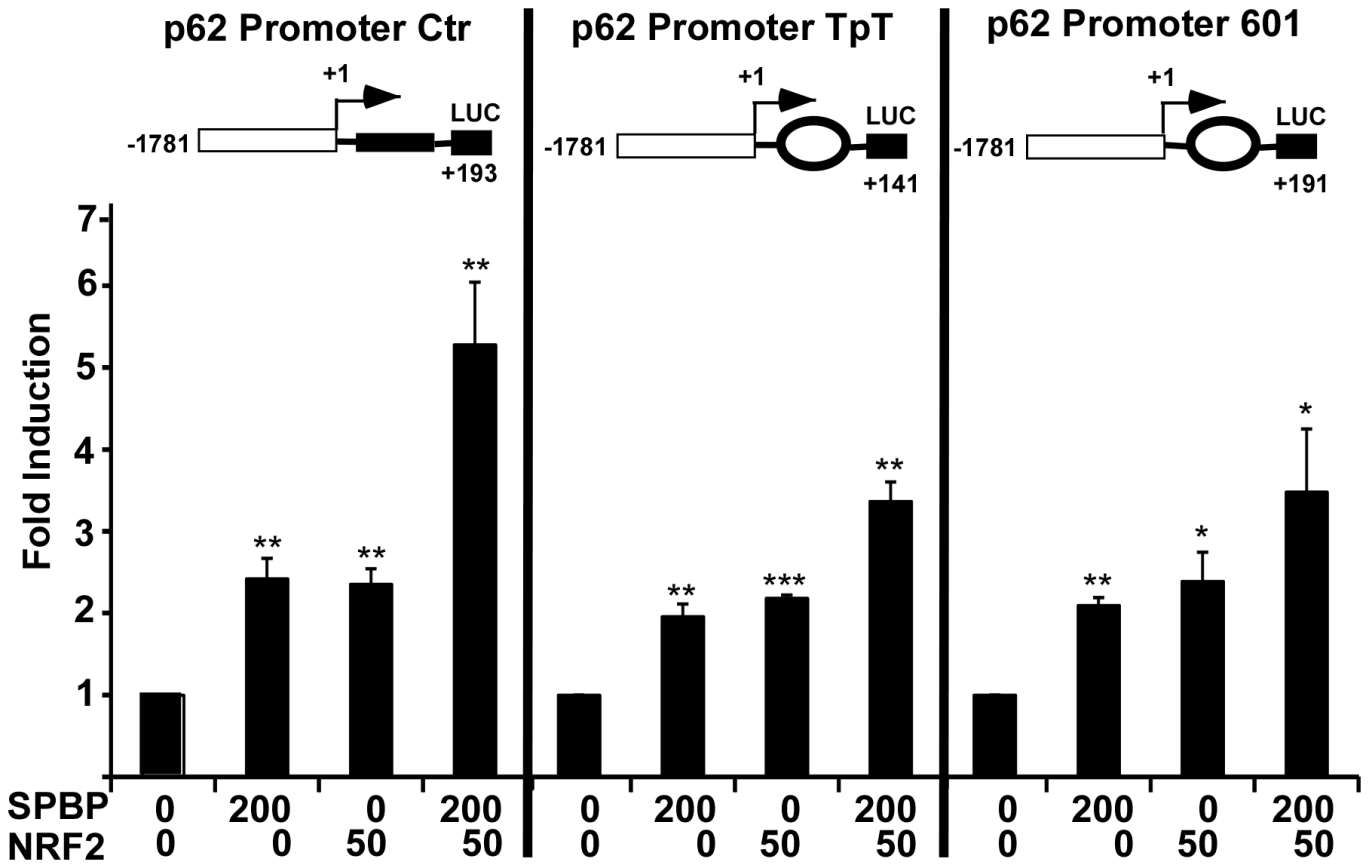
Nucleosome positioning sequences impair the synergistic effect of NRF2 and SPBP on the p62 promoter. Reporter constructs (60 ng) containing the indicated nucleosome position sequences inserted downstream of the transcription start site of p62 promoter were transfected into HEK293 cells together with the indicated amounts of expression plasmids for SPBP and/or NRF2. The luciferase activity of the promoter constructs cotransfected with empty expression plasmids was set to 1. The data represent the mean of three independent experiments with standard deviations, each performed in triplicate (***p<0.001, **p<0.01, *p<0.05).

SPBP contains two nucleosome binding domains and displays strong affinity for nucleosomes [31]. Nucleosomes pose a barrier to RNA polymerase II, thus transcription through chromatin require the concerted action of transcriptional coactivators including ATP-dependent remodeling machines, histone modification enzymes and histone chaperones (reviewed in [38]). The ATP-dependent remodeling enzyme SWI/SNF travels with RNA polymerase II and evicts histones on active genes [39], and it is shown that the transcriptional coactivator and SWI-SNF chromatin-remodeling-complex protein BRG1 is recruited to positioned nucleosomes and facilitate RNA polymerase II traversal of nucleosomes during transcriptional elongation [40]. Knock-down of SWI/SNF by RNAi resulted in pausing of polymerase II at an artificial introduced 601 nucleosome positioning sequence. In order to investigate whether SPBP acted on the p62 promoter by facilitating transcription through positioned nucleosomes, we introduced two synthetically nucleosome positioning sequences into the p62 promoter-luciferase construct (Figure 4). The 601 DNA sequence [41] has a stronger tendency to form nucleosomes than the TpT sequence [42], [43]. As control, 147 base pairs of a prokaryotic ampicillin resistance gene was inserted in the same position as the nucleosome forming sequences [40]. Luciferase reporter gene assays showed that SPBP mediated enhancement of transcription initiated on the p62 promoter was slightly impaired by both the TpT and the 601 nucleosome positioning sequences (Figure 4 and Figure S1A). The NRF2 mediated activation of the p62 promoter was inhibited similarly (Figure 4 and Figure S1A). Importantly, the synergism of SPBP and NRF2 was clearly abolished by inserted nucleosome positioning sequences (Figure 4). These results suggest that nucleosome traversal during transcriptional elongation is not significantly facilitated by SPBP. Similar results were obtained using the NRF2 regulated promoter NQO1 with inserted nucleosome positioning sequences downstream of the transcriptional initiation site (Figure S1B). Both the SPBP and the NRF2 mediated transcriptional activation of the NQO1 promoter was inhibited by inserted nucleosome position sequences. Hence, SPBP seems to exert its coactivation function via another mechanism than facilitating RNA polymerase II traversal through a nucleosome barrier.

### SPBP is not an autophagy substrate

**Figure 5:**
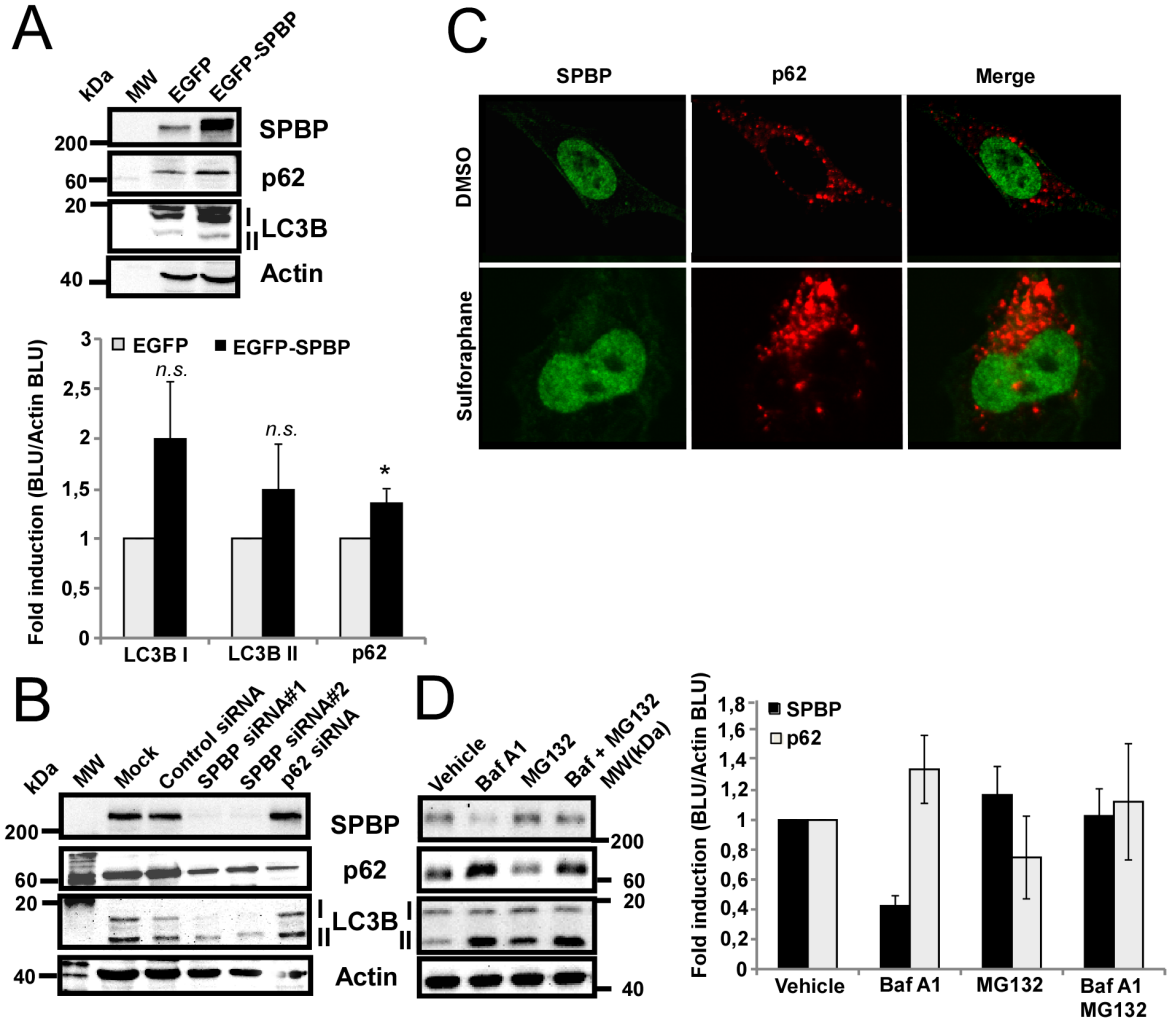
SPBP is not degraded by autophagy. (**A**) Over-expression of SPBP enhances the protein level of p62 in U2OS cells. Cell extracts of U2OS cells stably over-expressing EGFP or EGFP-SPBP were exposed to western blotting using the indicated antibodies. The graph shows fold induction calculated and correlated to actin in three independent experiments with standard deviations (*p<0.05, *n.s.* not significant). (**B**) siRNA mediated knock-down of SPBP impairs LC3B expression. Cell extracts of HeLa cells transfected with SPBP siRNA, p62 siRNA or Control siRNA were subjected to western blotting using the indicated antibodies. (**C**) Endogenous SPBP is not recruited to p62 bodies upon sulforaphane treatment. HeLa cells were treated with DMSO (upper panel) or 20 µM sulforaphane for 8 hours (lower panel), fixed and stained with antibodies against SPBP (green) and p62 (red). (**D**) SPBP is degraded by the proteasome. Extracts from HeLa cells starved in HBSS (2 hours), left untreated or treated with Bafilomycin A1 (0.2 µM) or/and MG132 (0.2 µM) for 4 hours were subjected to western blot using the indicated antibodies. The graph shows the average quantification of the expression levels of p62 and SPBP obtained in two independent experiments.

We have established an U2OS cell line stably over-expressing EGFP-SPBP [27]. Western blot on cell extracts from these cells shows that there is a significant 1.4 fold enhancement of p62 protein expression in U2OS cells overexpressing EGFP-SPBP compared to cells overexpressing EGFP only (Figure 5A). Since p62 is both an autophagic receptor and an autophagic substrate, the increased level of p62 could be due to inhibited degradation by autophagy. However, western blot of the autophagic marker protein LC3B did not show any reduced formation of LC3B II in these cells (Figure 5A). Furthermore, treatment of the cells with the drug Bafilomycin A1 resulted in increased protein levels of p62 and LC3B II, indicating that p62 is degraded normally by autophagy in the stably transfected U2OS cells (Figure S2A). Bafilomycin A1 acts as an autophagy inhibitor as it prevents maturation of autophagic vacuoles by inhibiting fusion between autophagosomes and lysosomes. Collectively, these results indicate that increased amounts of p62 in U2OS cells overexpressing SPBP is due to enhanced expression of p62, and not inhibition or impairment of the autophagy process. Moreover, siRNA mediated depletion of SPBP in HeLa cells reproducibly reduced the expression levels of LC3B I and reduced LC3B II formation (Figure 5B and Figure S2B). This reduction was not due to the decreased levels of p62, since siRNA mediated knock-down of p62 had no impact on the LC3B protein level (Figure 5B and Figure S2B). All together, these results suggest that SPBP stimulates autophagy activity by enhancing the expression levels of LC3B and p62. We next raised the question whether SPBP itself could be degraded by autophagy. SPBP is a nuclear protein, and hence expected to be degraded by the proteasome. However, some nuclear proteins are reported to translocate to the cytoplasm and be involved in autophagy upon cellular stress, such as TP53INP1 [44], TP53INP2/DOR ([45], [46]) and HMGB1 [47]. Immunostaining of endogenous SPBP and p62 in HeLa cells did not indicate any co-localization of SPBP with p62 in p62 bodies (Figure 5C, upper panel), not even upon sulforaphane treatment for eight hours (Figure 5C, lower panel). Furthermore, immunoprecipitation of SPBP from HeLa cell extracts did not co-precipitate detectable amounts of p62 (data not shown). This suggests that SPBP do not translocate to the cytoplasm upon sulforaphane treatment. To further analyze whether SPBP could be recruited to autophagosomes and degraded by autophagy, the stability of SPBP was determined in HeLa cells which were amino acid starved and treated with the autophagy inhibitor Bafilomycin A1 or the proteasome inhibitor MG 132 for 4 hours. The results presented in Figure 5D show that the amount of SPBP decreases when autophagy is inhibited, but not when the proteasome is inhibited. This clearly indicate that SPBP is mainly degraded by the proteasome.

### SPBP acts as a coactivator of NRF2

**Figure 6:**
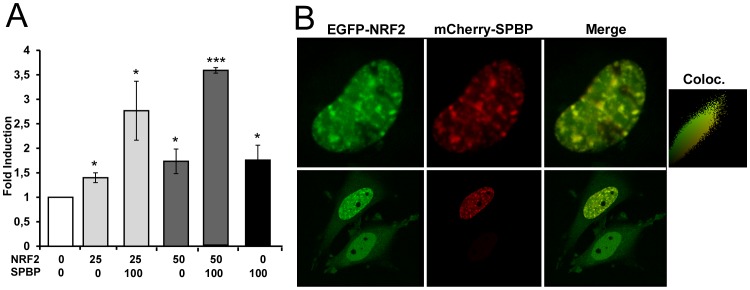
SPBP and NRF2 cooperate to induce expression from the p62 promoter, and colocalize in nuclear speckles. (**A**) SPBP and NRF2 cooperate to enhance expression from the wild type p62 promoter. Transient transfections were carried out in HEK293 cells using 60 ng of the p62 promoter construct (−1781/+46), and 50 ng or 100 ng of an NRF2 expression vector, and 100 ng of a SPBP expression vector, as indicated. The data represent the mean of three independent experiments with standard deviations, each performed in triplicate (***p<0.001, *p<0.05). (**B**) SPBP recruits NRF2 to specific nuclear speckles. HeLa cells were transiently transfected with expression vectors for EGFP-NRF2 and mCherry-SPBP, and analysed 24 hours post transfection by live cell imaging using a confocal laser scanning fluorescence microscope. The Pearson's colocalisation scatter was generated using Volocity (Perkin Elmer).

Reporter gene assays using the wild type p62 promoter indicated that coexpression of SPBP and NRF2 results in a synergistic activation (Figure 6A). To further explore if SPBP may act as a coactivator of NRF2, we analyzed whether the two proteins colocalize in cells. SPBP is a chromatin binding protein enriched in chromatin rich regions in the cell nucleus [31]. NRF2 is under normal conditions kept in the cytoplasm and directed to degradation by interaction with the molecular sensor protein KEAP1. Upon oxidative stress, NRF2 is released from KEAP1 and translocates to the nucleus where it can bind to ARE elements and direct transcription of p62, phase II enzymes and other stress induced proteins with ARE elements in their control regions (reviewed in [48]). Coexpressed EGFP-NRF2 and Cherry-SPBP in HeLa cells displayed nearly complete colocalization in the nucleus, with a calculated Pearson's correlations coefficient of around 0.95 for all cells in which they were coexpressed (Figure 6B). When Cherry-SPBP was present, EGFP-NRF2 was recruited to the nuclear speckles enriched for SPBP. In contrast, cells without Cherry-SPBP expression displayed a diffuse nuclear EGFP-NRF2 staining (Figure 6B, lower panel, and Figure S2C). This indicates that SPBP has the ability to recruit NRF2 to specific locations on chromatin. However, immunoprecipitation experiments in HeLa cells revealed that SPBP coprecipitate very weakly with NRF2 (Figure S2D), suggesting that their co-localization is dependent on chromatin. Interestingly, Western Blots of NRF2 in HeLa cell extracts treated with SPBP siRNA and sulforaphane showed that the amount of NRF2 is significantly reduced when SPBP is knocked down (Figure S2E). The KEAP1 expression level on the other hand, is unaffected by SPBP siRNA treatment, indicating that the reduced NRF2 expression is not due to enhanced degradation mediated by KEAP1. Collectively, these results suggest that SPBP acts as a coactivator of NRF2, promoting expression of NRF2 and NRF2 target genes such as p62 and LC3B under oxidative stress conditions mimicked by sulforaphane treatment.

## Discussion

Here we have shown that the expression of the transcriptional coregulator SPBP is upregulated upon sulforaphane treatment of cells, and that SPBP may be involved in the protection of cells against oxidative stress since it acts as a coactivator of NRF2. Interestingly, a recent genome wide siRNA screen showed that SPBP (named TCF20 therein) was important for both Cyp1A1 and NQO1 expression upon cellular stress induced by 2,3,7,8-tetrachlorodibenzo-p-dioxin [49]. Induction of NQO1 is previously shown to be completely dependent on the NRF2-ARE pathway [50]. This clearly supports our results presented here, and adds to an increasing amount of reports regarding stress-mediated regulation of transcriptional coregulators and their impact on stress-induced cellular processes. Many of these cofactors have the ability to act as sensors for metabolic or oxidative changes within the cells, inducing expression of genes involved in cell adaption to the changed metabolic or oxidative conditions. Examples are; i) the chromatin remodeling factor Pontin which is methylated in hypoxic condition, resulting in strongly activation of a subset of hypoxia target genes via HIF-1α [51], ii) the chromatin remodeling factor Reptin, which also is methylated in hypoxic conditions, and negatively regulate a subset of hypoxia-responsive genes [52], iii) the level and enzymatic activity of the histone metyltransferases and transcriptional regulators G9a, Suv39h and PRMT2 which are shown to be regulated by hypoxia (reviewed in [53]), and importantly, the expression levels of several members of the transcriptional coregulator family JmjC (demethylases) which are shown to be induced by hypoxia (reviewed in [53]). Their enzymatic activity requires molecular oxygen, making them perfect as oxygen sensors. In addition, expression of the Class III histone deacetylase Sirt1 is induced by E2F1 phosphorylated by the stress-responsive kinase ataxia telangiectasia mutated (ATM), by p53 and FOXO3a (activated in nutrient-deprived mammalian cells), by c-Myc and by the redox sensor carboxy terminal of E1A-binding protein (CtBP) (reviewed in [54]). Expression of the coactivator PGC-1α is induced by beta guanidinopropionic acid (GPA) in striatum and cerebral cortex [55], while the transcription coactivator Eya2 is shown to be upregulated during physiological hypertrophy [56]. Hence, transcriptional coregulators are important for cell adaption and protection against oxidative stress, and especially for the prolonged cellular adaption since their impact are mainly at the transcriptional level. SPBP is itself induced upon sulforaphane treatment and thus may be important for the prolonged upregulation of sulforaphane induced genes such as NRF2, p62, LC3B, NQO1 and other phase II detoxification enzymes.

It is well known that the transcription factor NRF2 regulates the basal and inducible expression of a wide array of antioxidant genes via AREs in their control regions (reviewed in [1], [2], [57]). We have shown that NRF2 binds to a specific ARE element in the autophagic receptor protein p62 promoter and increases its expression level upon sulforaphane treatment of cells [25]. Here we have shown that the coactivator SPBP enhances expression from the p62 promoter via the ARE elements in its promoter control region. siRNA mediated knock-down of SPBP significantly reduced both the amount of p62 proteins and the number of p62 bodies in HeLa cells. Furthermore, we found that NRF2 and SPBP act synergistically on the p62 promoter, and that knock-down of SPBP significantly reduced the sulforaphane induced expression of NRF2. Interestingly, it is reported that NRF2 contains two ARE-like elements in its own promoter and autoregulate its own expression via the ARE-like element located at position −754 [58]. Furthermore, NRF2 is found to be associated with its own promoter in genome wide chromatin immunoprecipitation analyses (ENCODE project at www.genome.gov). This suggests that SPBP may enhance NRF2 induction via the ARE elements in the NRF2 promoter, and thereby strengthen both the magnitude and the duration of the sulforaphane mediated enhancement of NRF2 expression – and the magnitude and the duration of expression of proteins regulated by NRF2 such as p62. From this we propose that SPBP may be a player in the positive feedback loop established to enhance expression of NRF2 and NRF2 regulated genes in response to oxidative stress, contributing to the strength and duration of the oxidative stress response. Additionally, we found that over-expression of SPBP recruits NRF2 to specific nuclear speckles previously identified as chromatin-rich regions [31]. This indicates that SPBP may recruit NRF2 to specific regions on chromatin, even if co-immunoprecipitation analysis indicated very weak association between NRF2 and SPBP. The histone acetyltranseferase MOZ is shown to act as a coactivator of NRF2-MafK. MOZ was found to bind to MafK via its PHD domain [59]. This suggests that SPBP may recruit NRF2 to chromatin via its ePHD/ADD domain interacting with MafK. This should be addressed in further studies.

Others have shown that the DNA binding activity of NRF2 is modulated by the transcriptional coregulator CBP/p300 [34]. CBP/p300 directly acetylates lysine residues in the DNA binding domain of NRF2 in response to arsenite-induced stress, resulting in enhanced DNA binding and enhanced transcription of NRF2 regulated genes. Here we found that CBP clearly enhances expression from the p62 promoter. However, the enhancement seemed not to be dependent on the ARE elements in the promoter. This suggests that CBP is able to mediate effect on the p62 promoter at other sites than the two conserved ARE elements mutated here.

We observed that depletion of SPBP impaired expression of LC3B. Hence, SPBP may have impact on the autophagy process by enhancing expression of LC3B, leading to increased formation of LC3B II. Recent results presented by Fujita et al. [60], indicate that siRNA mediated knock down of NRF2 significantly reduces LC3B expression. Furthermore, sequence analysis of the LC3B promoter predicts two putative NRF2 binding sites, one located around position −500 and one located further upstream around position −1750. Interestingly, these locations of NRF2 binding sites in the LC3B promoter are similar to the locations of the two NRF2 binding sites in the p62 promoter. Thus, SPBP may regulate LC3B expression via these NRF2 binding sites similarly as we here have shown for the p62 promoter. An interesting question to address for further study would therefore be whether SPBP together with NRF2 is involved in stress-induced coregulation of LC3B and p62, which both are important proteins in selective autophagy.

So far, no enzymatic activity has been associated with SPBP, and bioinformatic analyses do not predict any specific domains or regions with similarity to enzyme activity. Hence, the mechanism involved in SPBP induced enhancement of ARE containing promoters is unclear. We have previously shown that SPBP binds strongly to nucleosomes and is able to bind histones directly [31]. Nucleosomes pose a barrier to RNA polymerase II, and it has been shown that some transcriptional coactivators enhance transcription by facilitating RNA polymerase II traversal of nucleosomes during transcriptional elongation [40]. Here we evaluated whether SPBP had the ability to facilitate transcription when a nucleosome forming sequence was inserted immediately downstream of the transcription initiation site in the p62 and NQO1 promoters. We found that the SPBP mediated transcriptional stimulation was reduced when a nucleosome positioning sequence was present. The NRF2 mediated induction of the p62 promoter was reduced similarly. Importantly, coexpression of SPBP and NRF2 did not result in synergistic stimulation of the p62 promoter constructs with a positioned nucleosome sequence, indicating that SPBP do not act on the p62 promoter by facilitating traversal of a nucleosome structure. Similar results were obtained for the NQO1 promoter, which strengthen the conclusion.

p62 is an autophagy receptor protein degraded by autophagy. The observation that SPBP affected the expression level of p62 and the amount of p62 bodies in the cytoplasm, raised the question whether SPBP is directly involved in regulation of the autophagy machinery in the cell. The nuclear proteins HMGB1 [47], TP53INP1 [44], and DOR/TP53INP2 [45], [46] are reported to be translocated to the cytoplasm upon cellular stress and participate in regulation of autophagosome formation and protein degradation. However, we were not able to detect any nuclear translocation of SPBP upon sulforaphane treatment of HeLa cells. But, we found that SPBP has impact on the expression of proteins directly involved in autophagy, such as LC3B and p62, and on NRF2 that is involved in stress-induced expression of proteins involved in autophagy. However, whether NRF2 inhibits or stimulates the autophagy machinery is somehow contradictory in the literature (see f. ex. [61]–[63]). Here, we see a correlative upregulation of NRF2, p62, and SPBP within the first 12 hours of sulforaphane treatment, peaking at 6 – 10 hours of treatment. Another study has looked at protein expression levels after 24 hours or more with Mitoquinone treatment, and found that NRF2 downregulates LC3B expression [63]. This may indicate that as an immediate response to oxidative stress, NRF2 stimulates the autophagy machinery, while it downregulates autophagy activity in the prolonged response. To conclude, our data indicate that SPBP, which itself is induced by oxidative stress, is important for enhanced induction of proteins involved in the cellular defensive program against oxidative stress, such as NRF2, and the autophagy machinery here represented by p62 and LC3B.

## Supporting Information

Figure S1 **Nucleosome positioning sequences impair the SPBP mediated enhancement of the p62 and NQO1 promoters.** (**A and B**) Reporter constructs (60 ng) containing the indicated nucleosome position sequences inserted downstream of the transcription start site of p62 promoter (**A**) or NQO1 promoter (**B**) were transfected into HEK293 cells together with the indicated amounts of expression plasmids for SPBP or NRF2. The luciferase activity of the promoter constructs cotransfected with empty expression plasmid was set to 1. The data represent the mean of three independent experiments with standard deviations, each performed in triplicate.
(TIF)

Figure S2 **SPBP impacts on NRF2, p62 and LC3B expression levels in HeLa and U2OS cells.** (**A**) p62 seems to be degraded normally by autophagy in the U2OS cells over-expressing EGFP-SPBP. Cell extracts from U2OS cells overexpressing EGFP-SPBP or EGFP, and treated with DMSO or Bafilomycin A1 (0.2 µM for 4 hours), were separated by SDS-PAGE and blotted against the indicated antibodies. (**B**) Knock-down of SPBP reduces the expression levels of NRF2, p62 and LC3B in HeLa cells. Cell extracts of HeLa cells transfected with the indicated siRNAs for 48 hours, and stimulated by sulforaphane (20 µM) or DMSO for the last 8 hours, were separated by SDS-PAGE and blotted against the indicated antibodies. (**C**) NRF2 is not recruited to specific nuclear speckles when coexpressed with mCherry. HeLa cells were transiently transfected with expression vectors for EGFP-NRF2 and mCherry, and analysed 24 hours post transfection by live cell imaging using a confocal laser scanning fluorescence microscope. Pearson's colocalisation scatter was generated using Volocity (Perkin Elmer). (**D**) SPBP associates weakly with NRF2. HeLa cells were transfected with expression vectors for EGFP-NRF2 and Myc-SPBP, or EGFP and Myc-SPBP. EGFP-NRF2 was immunoprecipitated with GFP antibody 20 hours post transfection. Precipitated EGFP-NRF2 and co-precipitated SPBP were detected by western blotting using the indicated antibodies. (**E**) siRNA mediated knock-down of SPBP impairs sulforaphane induced NRF2 expression. HeLa cells were transfected with SPBP siRNAs or Control siRNA as indicated. Cells were treated with sulforaphane for eight hours two days post transfection. The cell extracts were subjected to western blot using the indicated antibodies. The graph shows fold reduction calculated and correlated to actin in two independent experiments with standard deviations.
(TIF)
