# Supplementary figures and images for: SPBP Is a Sulforaphane Induced Transcriptional Coactivator of NRF2 Regulating Expression of the Autophagy Receptor p62/SQSTM1
Source: PLoS One. 2014 Jan 9;9(1):e85262. doi: 10.1371/journal.pone.0085262 (PMC3887019; doi:10.1371/journal.pone.0085262)

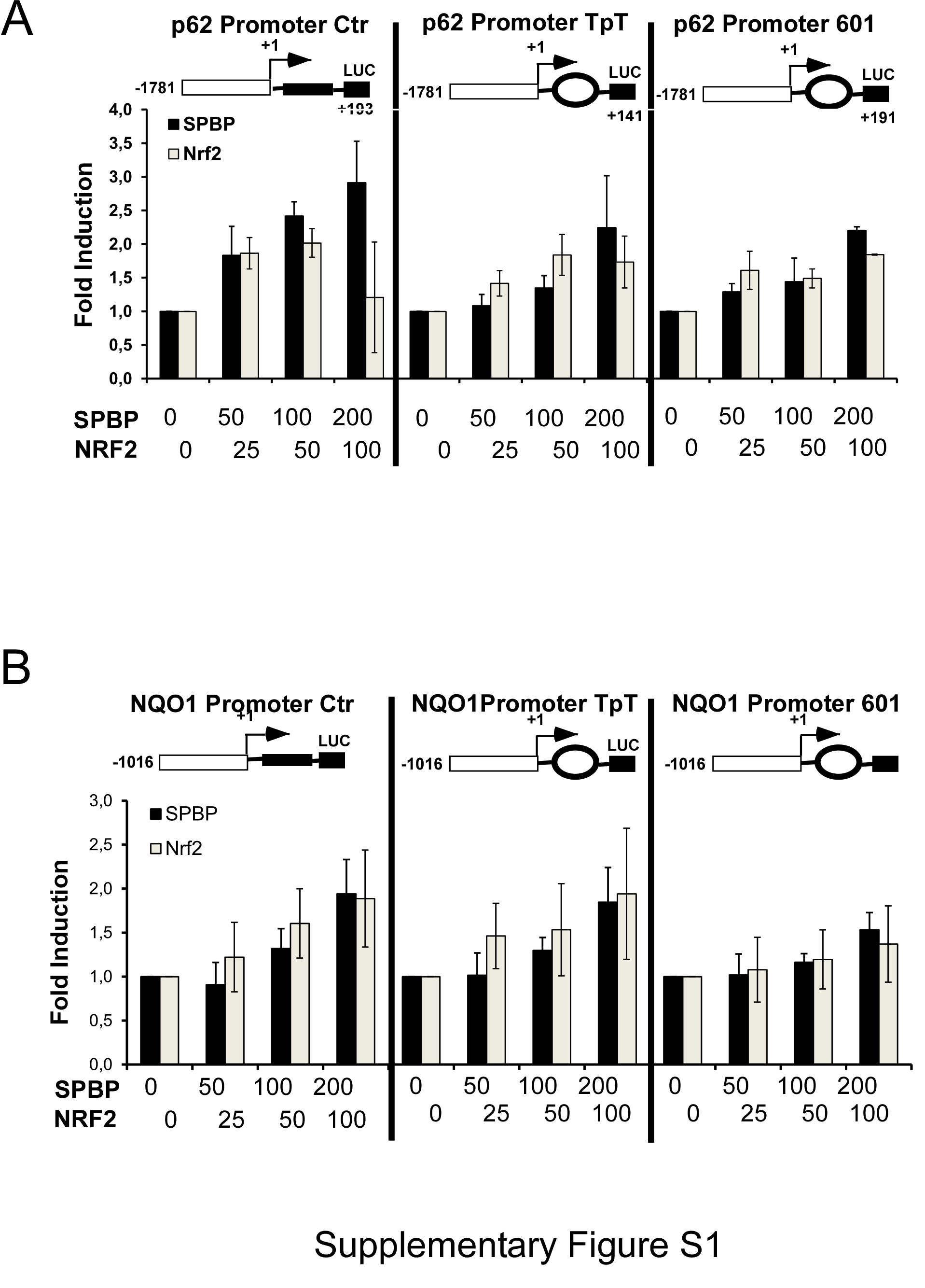

Supplement: Figure S1 — Nucleosome positioning sequences impair the SPBP mediated enhancement of the p62 and NQO1 promoters. (A and B) Reporter constructs (60 ng) containing the indicated nucleosome position sequences inserted downstream of the transcription start site of p62 promoter (A) or NQO1 promoter (B) were transfected into HEK293 cells together with the indicated amounts of expression plasmids for SPBP or NRF2. The luciferase activity of the promoter constructs cotransfected with empty expression plasmid was set to 1. The data represent the mean of three independent experiments with standard deviations, each performed in triplicate. (TIF) [file pone.0085262.s001.tif]

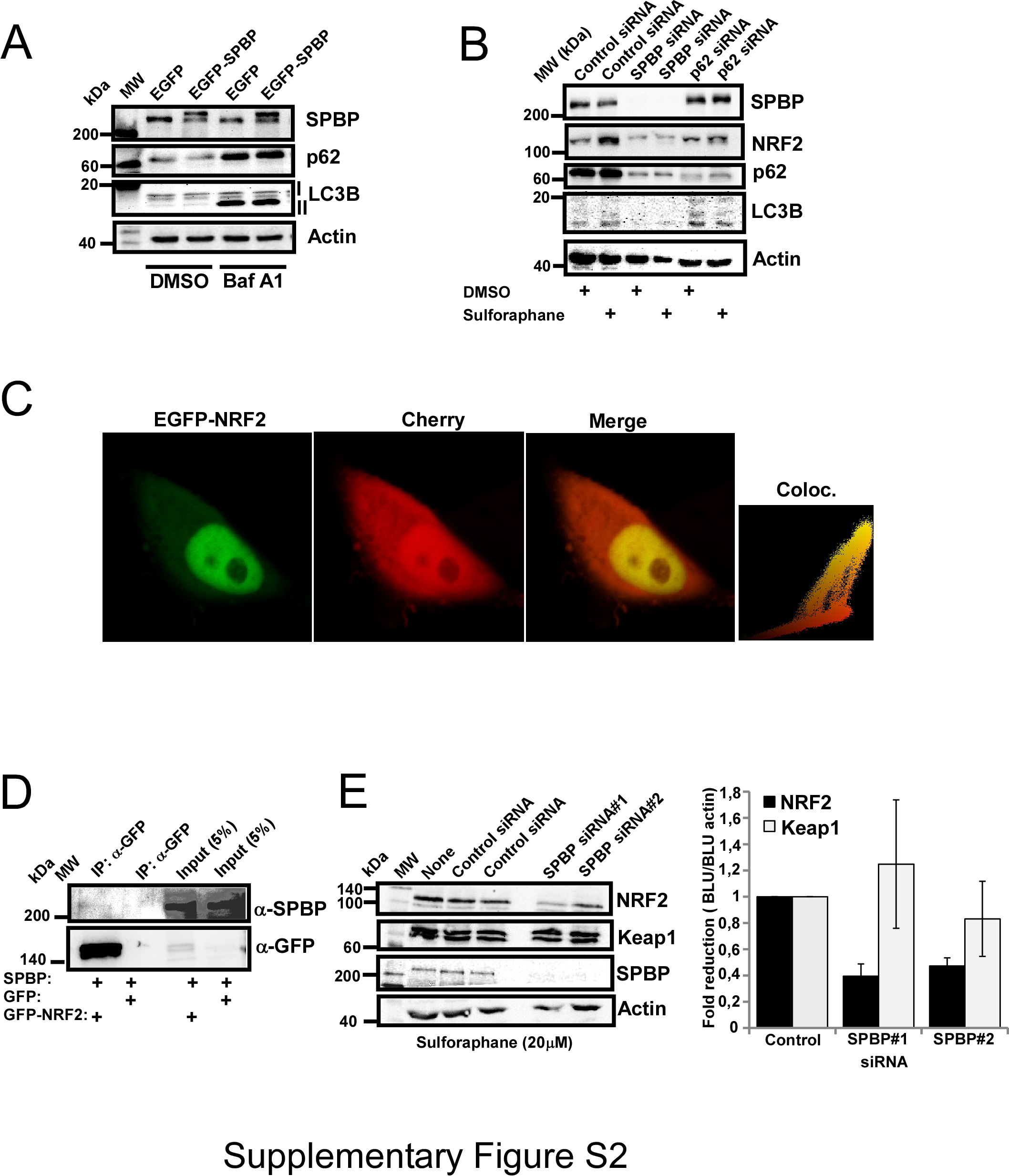

Supplement: Figure S2 — SPBP impacts on NRF2, p62 and LC3B expression levels in HeLa and U2OS cells. (A) p62 seems to be degraded normally by autophagy in the U2OS cells over-expressing EGFP-SPBP. Cell extracts from U2OS cells overexpressing EGFP-SPBP or EGFP, and treated with DMSO or Bafilomycin A1 (0.2 µM for 4 hours), were separated by SDS-PAGE and blotted against the indicated antibodies. (B) Knock-down of SPBP reduces the expression levels of NRF2, p62 and LC3B in HeLa cells. Cell extracts of HeLa cells transfected with the indicated siRNAs for 48 hours, and stimulated by sulforaphane (20 µM) or DMSO for the last 8 hours, were separated by SDS-PAGE and blotted against the indicated antibodies. (C) NRF2 is not recruited to specific nuclear speckles when coexpressed with mCherry. HeLa cells were transiently transfected with expression vectors for EGFP-NRF2 and mCherry, and analysed 24 hours post transfection by live cell imaging using a confocal laser scanning fluorescence microscope. Pearson's colocalisation scatter was generated using Volocity (Perkin Elmer). (D) SPBP associates weakly with NRF2. HeLa cells were transfected with expression vectors for EGFP-NRF2 and Myc-SPBP, or EGFP and Myc-SPBP. EGFP-NRF2 was immunoprecipitated with GFP antibody 20 hours post transfection. Precipitated EGFP-NRF2 and co-precipitated SPBP were detected by western blotting using the indicated antibodies. (E) siRNA mediated knock-down of SPBP impairs sulforaphane induced NRF2 expression. HeLa cells were transfected with SPBP siRNAs or Control siRNA as indicated. Cells were treated with sulforaphane for eight hours two days post transfection. The cell extracts were subjected to western blot using the indicated antibodies. The graph shows fold reduction calculated and correlated to actin in two independent experiments with standard deviations. (TIF) [file pone.0085262.s002.tif]
